# Near-field infrared nanoscopic study of EUV- and e-beam-exposed hydrogen silsesquioxane photoresist

**DOI:** 10.1186/s40580-022-00345-3

**Published:** 2022-12-02

**Authors:** Jiho Kim, Jin-Kyun Lee, Boknam Chae, Jinho Ahn, Sangsul Lee

**Affiliations:** 1 Pohang Accelerator Laboratory, POSTECH, Pohang, 37673 Republic of Korea; 2 Department of Polymer Science and Engineering, Inha University, Incheon, 22212 Republic of Korea; 3 Division of Materials Science and Engineering, Hanyang University, Seoul, 04763 Republic of Korea

**Keywords:** Nanoscale chemical visualization, Hydrogen silsesquioxane (HSQ), Infrared nanoscopy, Scattering-type scanning near-field optical microscope (s-SNOM), Photoreaction, Photoresist, Photoresist metrology

## Abstract

This article presents a technique of scattering-type scanning near-field optical microscopy (s-SNOM) based on scanning probe microscopy as a nanoscale-resolution chemical visualization technique of the structural changes in photoresist thin films. Chemical investigations were conducted in the nanometer regime by highly concentrated near-field infrared on the sharp apex of the metal-coated atomic force microscopy (AFM) tip. When s-SNOM was applied along with Fourier transform infrared spectroscopy to characterize the extreme UV- and electron-beam (e-beam)-exposed hydrogen silsesquioxane films, line and space patterns of half-pitch 100, 200, 300, and 500 nm could be successfully visualized prior to pattern development in the chemical solutions. The linewidth and line edge roughness values of the exposed domains obtained by s-SNOM were comparable to those extracted from the AFM and scanning electron microscopy images after development. The chemical analysis capabilities provided by s-SNOM provide new analytical opportunities that are not possible with traditional e-beam-based photoresist measurement, thus allowing information to be obtained without interference from non-photoreaction processes such as wet development.

**Supplementary Information:**

The online version contains supplementary material available at 10.1186/s40580-022-00345-3.

## Introduction

Since the mass production of 7-nm-node logic devices first began in 2019, extreme ultraviolet lithography (EUVL) has made substantial advancements toward next-generation technology nodes, thereby enabling the manufacture of 5-nm node devices. Going a step further, the international roadmap for devices and systems (IRDS™) has announced that it expects 3-nm nodes in 2022 and 1.5-nm nodes in 2028 [1]. According to IRDS™, 1.5-nm-node technology requires 8 nm half-pitch (HP) patterns and a line edge roughness (LER) value under 1.2 nm. These expectations could only be achieved with progress in photoresist technology that provides reliable stencil materials for the construction of electric circuits on semiconductor substrates.

The performance of EUV photoresist is mainly characterized by three parameters: sensitivity, resolution, and line-edge roughness (LER). There is a tradeoff relationship among these parameters, which is mainly caused by the photon shot-noise effect and additionally aggravated by chemical stochastic issues, thus necessitating the development of ingenious strategies to overcome these limitations to achieve high-performance EUV [2–14].

Further, as the minimum feature size of photoresist patterns has approached the sub-10 nm regime, it has become increasingly necessary to use extremely thin photoresist films. Failing to do so would likely lead to photoresist patterns with a big aspect ratio that would collapse during the development process. In general, patterns with an aspect ratio of 2:1 or less are recommended for photoresists, which means that the film thickness needs to be maintained within 10–20 nm [15]. This challenge of preventing pattern collapse has motivated foundational research into photoresist materials, leading to studies on various materials ranging from molecular photoresists to inorganic metal oxide photoresists (MOR) and, more recently, dry photoresists [15–28].

To date, most studies aiming to evaluate photoresist performance have done so by observing the physical patterns of lines and spaces after the exposure and development steps. With this type of research methodology, it is difficult to confirm whether the deformation or collapse of the photoresist pattern is caused by the exposure process or the pattern development conditions. As a result, there is a need for an adequate analysis method that can be used to distinguish between the causes. For example, if the latent image formed by exposure can be visualized before the development step, then the negative impact of pattern development can be estimated by comparing the before and after images. Further, the information on the pattern before development can be used for basic material research, such as that examining the changes in the photoresist chemical structure due to exposure, acid diffusion, and materials stochastic effects.

To evaluate the performance of photoresists, scanning electron microscopy (SEM) provides excellent surface information with nanoscale spatial resolution. However, SEM images are not suitable for the pre-development inspection of latent photoresist images because they only provide morphological information. In addition, as the film thickness becomes thinner, the image contrast of SEM is often insufficient to estimate the patterning performance of photoresists [29–33]. On the other hand, although FTIR spectroscopy, Raman spectroscopy, and X-ray photoelectron spectroscopy (XPS) can provide information on chemical compositions and structures, they cannot be used for pattern inspection due to their poor spatial resolution. To overcome this limitation, a state-of-the-art infrared approach with a nanoscale spatial resolution of 10 nm has been developed that allows for the visualization of the spectroscopic information of photoresists. Scattering-type scanning near-field optical microscopy (s-SNOM)—a branch of nano-IR techniques—is an optical AFM method that focuses infrared rays on the apex of a metal-coated AFM tip to generate a near-field IR within tens of nanometers [34–39]. Because s-SNOM is based on both scanning probe microscopy (SPM) and infrared interferometry, it may be used in material analysis in a wide range of material research fields [40–45]. Further, it does not suffer from low image contrast issues due to the thickness of the thin films [30, 46, 47].

In this study, we introduce s-SNOM as a potential investigation tool to be used in the study of photoresists. As s-SNOM is sensitive to the chemical absorptions of samples with a 10 nm resolution, it can be used for both the pattern inspection and the quantitative chemical analysis of the latent images drawn on photoresist films before a development process. To this end, hydrogen silsesquioxane (HSQ)—a standard photoresist for e-beam lithography—was cast onto thin films, which were subsequently exposed to EUV with doses of 10, 20, 40, 75, and 150 mJ/cm^2^. The 150 nm thick film was inspected using FTIR and s-SNOM, and the results were compared to show that s-SNOM has the capability to visualize the latent images. Another pair of HSQ films was exposed to a focused e-beam to inscribe half-pitch (HP) 100, 200, 300, and 500 nm line and space patterns. Then, one film of the pair was observed by s-SNOM with a particular energy corresponding to the characteristic absorption of the cage and network structure whereas the other film was developed and inspected by AFM. Using this method, the line and space patterns could be successfully visualized before and after a development step, and their linewidth and LER were analyzed.

## Methods/experimental

### Preparation of films

Double-sided polished silicon wafers with a specific resistivity (1–10 Ω cm) were used as substrates for IR measurement. To remove moisture from the surface, pre-baking was performed at 180 °C for 2 h. Next, the 150 nm thick HSQ (XR-1541 6%, in a methyl isobutyl ketone (MIBK) solvent, Dow Corning) film was coated by a spin coater (Spin-1200D, MIDAS SYSTEM), first at 500 rpm for 5 s to initially dispense the HSQ solution and then at 3500 rpm for 2 min for coating. The coated film was finally soft-baked at 180 °C for 2 min to remove the residual solvent. EUV exposure was performed with the high harmonic generated (HHG) EUV source (EUVO, EUV Source Generation System, FINE SEMITECH CORP.) at dose densities of 10, 20, 40, 75, and 150 mJ/cm^2^ with 12 × 12 μm^2^ transmission-type multiple slits. Line and space patterns with periods of 100, 200, 300, and 500 nm were engraved by a precise e-beam lithography system (ELS-7000, Elionix) under an electron density of 50 μC/cm^2^. The area of each pattern group was set to 12.5 × 50 μm^2^, and pattern groups were adjacent to each other so that the size of the whole pattern was 50 × 50 μm^2^. The development was conducted for 1 min with a 2.38% tetramethylammonium hydroxide (TMAH) aqueous solution (AZ 300MIF Developer, Merck).

### Measurements

The FTIR spectroscopic and s-SNOM measurements were taken on a 12D infrared spectroscopy (IRS) beamline, Pohang Accelerator Laboratory. The FTIR measurement was performed in transmission mode on an IR microscope (Hyperion 2000) added to a VERTEX 80v spectrometer (Bruker, GmbH) with a knife-edge aperture. FTIR spectra were obtained by Bruker's proprietary software, OPUS 8.1, with a 4 cm^−1^ spectral resolution. The s-SNOM measurement was conducted in near-field imaging mode (NIM) using the neaSNOM (neaspec GmbH). The specific infrared wavenumbers of 1125 cm^−1^ and 1067 cm^−1^ were chosen by a quantum cascade laser (QCL, MIR Cat, Daylight solutions). A Platinum/Iridium-coated AFM tip (Arrow-NCPt, Nanoworld) was used for the s-SNOM tapping mode scan with a tapping amplitude of 90 nm and a frequency *Ω* of 270 kHz. Tapping mode AFM measurement was also performed by neaSNOM without an infrared laser. Gwyddion, an open-source SPM software, was used for leveling and denoise correction for s-SNOM data and leveling correction for AFM data [48]. The linewidth and LER values of each pattern were measured by Lacerm, a freely available software, without any post-processing [49].

## Results and discussion

**Fig. 1:**
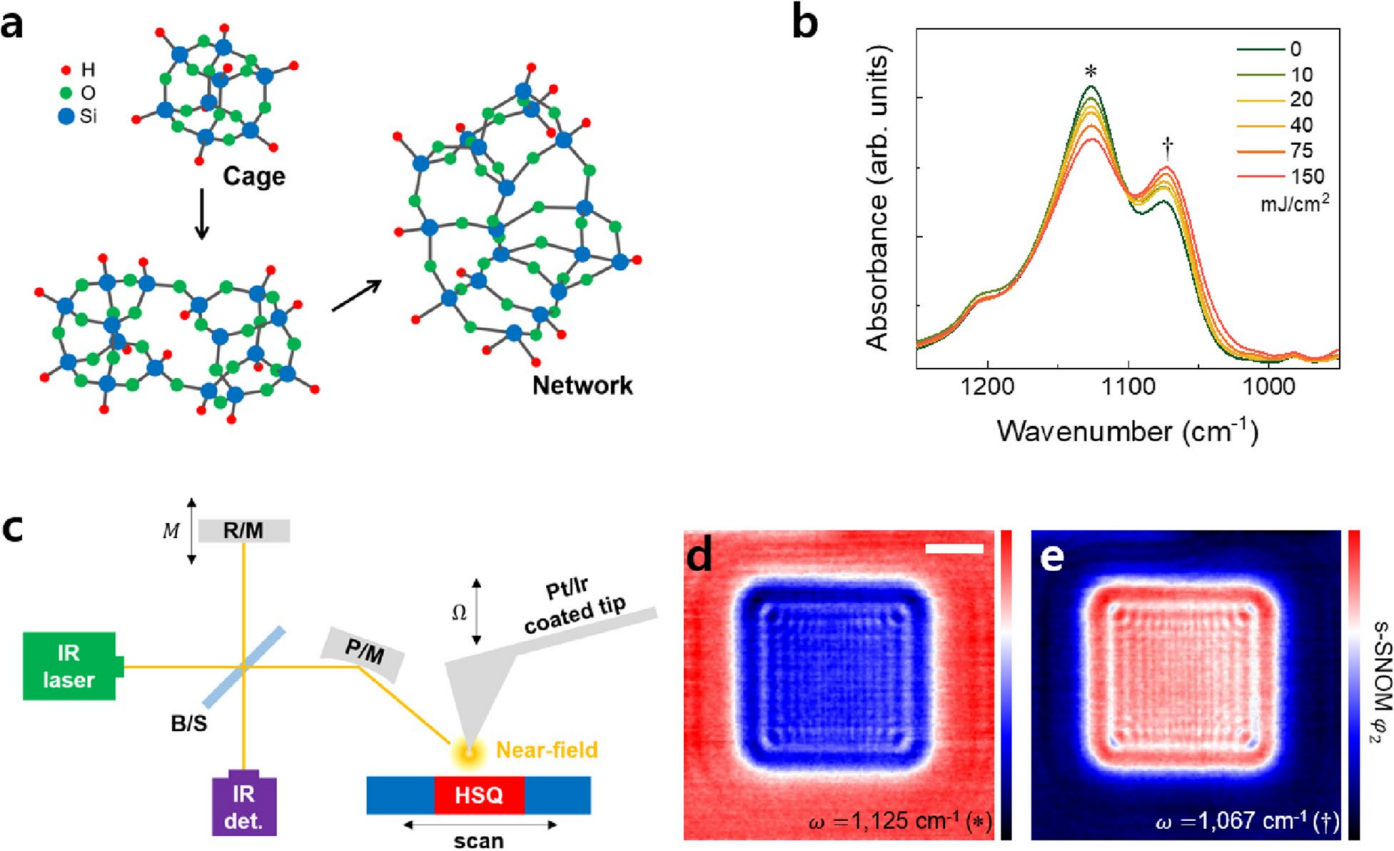
**a** Schematic illustration of the curing process of the hydrogen silsesquioxane (HSQ) resists from a cage-like structure to a network structure. **b** Fourier transform infrared (FTIR) spectra of as-cast and extreme ultraviolet (EUV)-exposed HSQ films for the range of Si–O–Si vibration. **c** Schematic illustration of scattering-type scanning near-field optical microscope (s-SNOM, beam splitter B/S, parabolic mirror P/M, reference mirror R/M). s-SNOM images of (**d**) cage-like structure (1125 cm^−1^) and **e** network structure (1067 cm^−1^) for 150 mJ/cm^2^. The red color indicates higher infrared absorption. The scale bar is 3 μm

Since its commercialization in the 1970s by Dow Corning, HSQ has been used as a negative tone photoresist as a standard e-beam resist along with poly (methyl methacrylate) (PMMA). As shown in Fig. 1a, pristine HSQ films mainly consist of cage-like (or cubic-like) [HSiO_3/2_] nanoclusters. When these cage-like nanoclusters are exposed to light, they react and lose hydrogen, subsequently making chemical bonds with neighboring nanoclusters to form cross-linked network structures. As the exposure energy increases, denser network structures are formed [50, 51]. Because the network structure has a lower solubility in a developing solution than the cage-like structure, HSQ operates as a negative tone photoresist. The chemical transitions from cage to network structures can be easily observed by IR and Raman spectroscopy, as these can track changes in specific molecular vibration, which are strongly related to the angle of Si–O bonding. Figure 1b shows the FTIR spectra of EUV-exposed HSQ thin films with the increasing energy of 10, 20, 40, 75, and 150 mJ/cm^2^. The intensities of the peak that are marked with an asterisk (1,125 cm^−1^) diminished as the high EUV exposure dose increased, indicating that the absorption strength of the cage-like structure decreased. Meanwhile, the increase in the peak marked with a dagger (1067 cm^−1^) indicates an increase in the proportion of network structures in HSQ. The same sample was also assessed using an s-SNOM measurement. Figure 1c depicts a schematic diagram of the s-SNOM setup, showing the Michelson interferometer, QCL infrared laser, and AFM system used to simultaneously obtain high-resolution optical and morphological information [34–37]. The beam splitter (B/S) divided the linear polarized infrared laser into sample and reference paths. The infrared laser passing along the sample path was focused on the end of the AFM tip by the parabolic mirror (P/M). The platinum-coated AFM tip had a very sharp apex, thus inducing a strong surface plasmon. Subsequently, the surface plasmon generated a strong electric field on the apex; as a result, s-SNOM could overcome the diffraction limit of the light and realize the 10 nm spatial resolution with a 10-micron-long infrared light. When the AFM tip was placed very close to the sample, near-field infrared interacted with the sample's surface and was scattered. The parabolic mirror focused the scattered light and sent it to the detector via a beam splitter. The infrared light heading for the reference path during this time was reflected by the reference mirror and directed to the detector. The metal tip and the reference mirror (R/M) vibrated at different frequencies of $\Omega$ and *M*, respectively. This detection mechanism—which receives two different frequencies and analyzes them to obtain the desired signal—is called Pseudoheterodyne detection. To increase detection efficiency, *M* is generally set to be ~ 100 times higher than $\Omega$. The Pseudoheterodyne detection method successfully detected both the amplitude (*s*) and phase ($\varphi$) of infrared, thus indicating that it simultaneously obtained the reflectance and the absorption of the sample [34, 52, 53].

Figure 1d, e shows s-SNOM 2nd order phase (${\varphi }_{2}$) mapping images for the cage-like structure (1125 cm^−1^) and the network structure (1067 cm^−1^), respectively. The color contrasts of the two images were fully inverted, thus reflecting the reversal of the ratio of the two structures. The exposed HSQ film has fewer cage-like structures and more network structures than the surrounding area. This caused the exposed area appeared to be darker than the surroundings in the cage structure mapping, and the exposed areas to be lighter than the surroundings in the network mapping. Further, the fringe patterns caused by the 0th diffraction light from the multiple silts that were used as a square aperture cannot be observed with conventional FTIR and AFM.

**Fig. 2:**
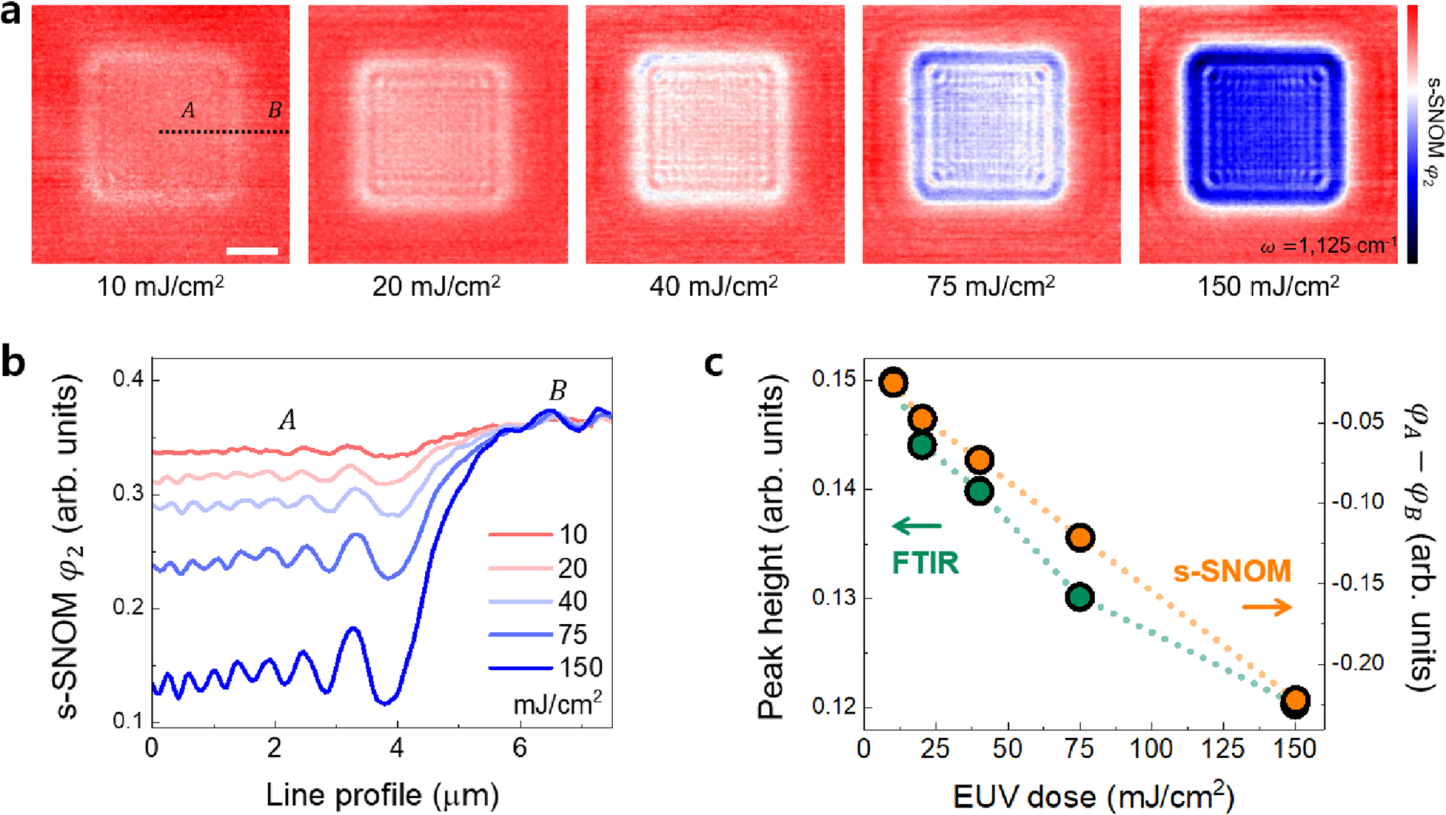
**a** EUV dose-dependent s-SNOM chemical imaging results. The red color indicates higher infrared absorption. The scale bar is 3 μm. **b** Line profiles taken from the image in (**a**) as indicated by the black dotted line. *A* and *B* respectively indicate EUV-exposed and non-exposed areas. **c** Comparison of the peak height of the cage-like structure from FTIR spectra and phase differences (${\varphi }_{A}-{\varphi }_{B}$) of s-SNOM at 1125 cm^−1^

Figure 2a shows s-SNOM ${\varphi }_{2}$ images, which are related to the absorption strength of the chosen laser energy. In this series, 1125 cm^−1^ was adopted, as it represented the relative chemical concentration of cage-like structures [50, 51]. As the exposure energy was increased, the relative ratio of the cage-like structures decreased, thus leading to deepening color contrasts between the EUV exposed (indicated as *A*) and non-exposed areas (*B*). For quantitative analysis, the line profile was plotted as shown in the black and dotted lines in Fig. 2a. Note that the s-SNOM provides relative optical signals within a single image for every individual scan; therefore, all profiles were plotted after the artificial leveling process. However, it did not indicate that the comparison of s-SNOM line profiles was irrelevant [41, 42]. Since the s-SNOM results shared the non-exposed area *B* in its border, this made it possible to compare absorption strength based on *B*. In other words, the infrared signal of area *B*—which is not affected by EUV exposure—could be used as a reference [54, 55]. As shown in Fig. 2c, the s-SNOM phase difference between areas *A* and *B* (${\varphi }_{A}-{\varphi }_{B}$) was linearly proportional to the EUV dose, which was consistent with the decreasing trend of the cage-like structure tracked by conventional FTIR.

Further, the advantage of the high-resolution nature of s-SNOM is evident in the results presented herein. Since we employed the transmission-type multiple slits as an aperture, the diffraction of light formed sub-micron lattice patterns on the photoresist. As can be seen in the phase images and line profiles, the s-SNOM with nanometer spatial resolution successfully visualized the lattice-form chemical structure with a hundred-nanometers spacing, which could not be obtained by FTIR imaging. This indicates that s-SNOM could potentially be used as a quantitative chemical analysis tool for photoresist characterization studies with nanometer spatial resolution.

**Fig. 3:**
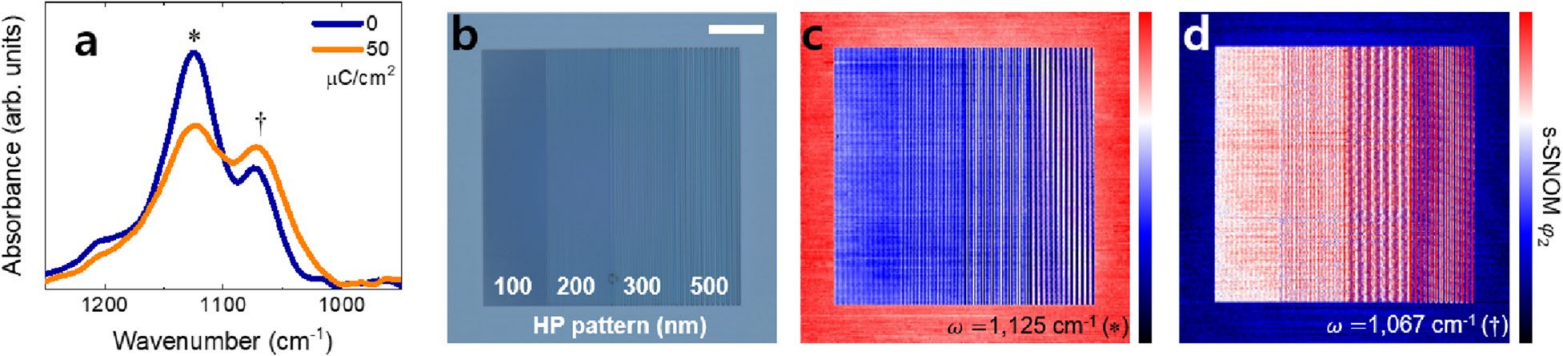
**a** FTIR spectra of as-cast (0 μC/cm^2^) and e-beam-exposed (50 μC/cm^2^) HSQ films for the range of Si–O–Si vibration. **b** Optical microscope image of e-beam patterned and developed HSQ. 100, 200, 300, and 500 nm periods with 1:1 line and space patterns can be seen. The imaging results of the s-SNOM phase (${\varphi }_{2}$) correspond to specific chemical structures. Images were taken at **c** 1125 cm^−1^ and **d** 1067 cm^−1^, and these respectively represent the cage-like and network structure. The red color indicates higher infrared absorption. The scale bar is 10 μm

The e-beam-exposed HSQ films showed similar chemical structure changes as the EUV-exposed films. The FTIR spectra for cage-dominant and network-dominant HSQ are shown in Fig. 3a. The blue spectrum was obtained with a pristine HSQ film whereas the orange spectrum came from the 50 μC/cm^2^ e-beam-exposed HSQ. Similar to the results of the EUV exposure experiment, the portion of the cake-like structure (1125 cm^−1^) decreased while that of the network structure increased (1067 cm^−1^). A pair of HSQ films was made using the same process to compare the pattern shapes before and after wet development. Following e-beam patterning, one sample was developed and inspected by an optical microscope and AFM; meanwhile, the other sample was investigated by s-SNOM without a wet development process.

Figure 3b shows an optical microscope image of the developed HSQ film. HP 100, 200, 300, and 500 nm lines and space patterns were observed. Figure 3c, d show the s-SNOM ${\varphi }_{2}$ images of the e-beam-inscribed HSQ film without development. As was the case in the EUV-irradiated results detailed above, the two structures—cage-like (Fig. 3c) at 1125 cm^−1^ and network (Fig. 3d) at 1067 cm^−1^—showed reversed trends. It was confirmed that the s-SNOM absorption imaging using these two inverted chemical images was also successful in e-beam-exposed HSQ.

**Fig. 4:**
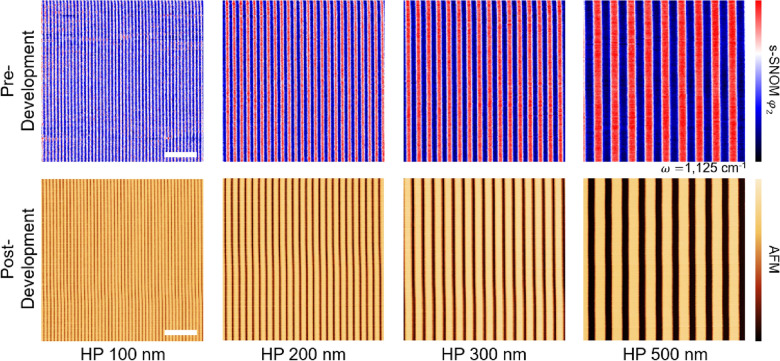
(Top) s-SNOM results of the pre-development chemical pattern visualization of latent images with the 1125 cm^−1^ infrared laser. The red color indicates higher infrared absorption. (bottom) Post-development pattern inspection of atomic force microscope (AFM) for half-pitch (HP) 100, 200, 300, and 500 nm patterns. The scale bar is 2 μm

Figure 4 shows two kinds of scanning images: The top row shows the pre-development pattern visualization images resulting from the s-SNOM chemical scan with 1125 cm^−1^ (cage scan). As expected, the exposed area (lines) with low cage absorption and the non-exposed area (spaces) with high cage absorption generated a periodic 1:1 pattern. Meanwhile, the images on the bottom row show the post-development pattern inspection results from conventional AFM topography. This successful matching of images proved that the pattern inspection could be achieved without the development process by visualizing the chemical spatial distribution of HSQ using s-SNOM.

**Fig. 5:**
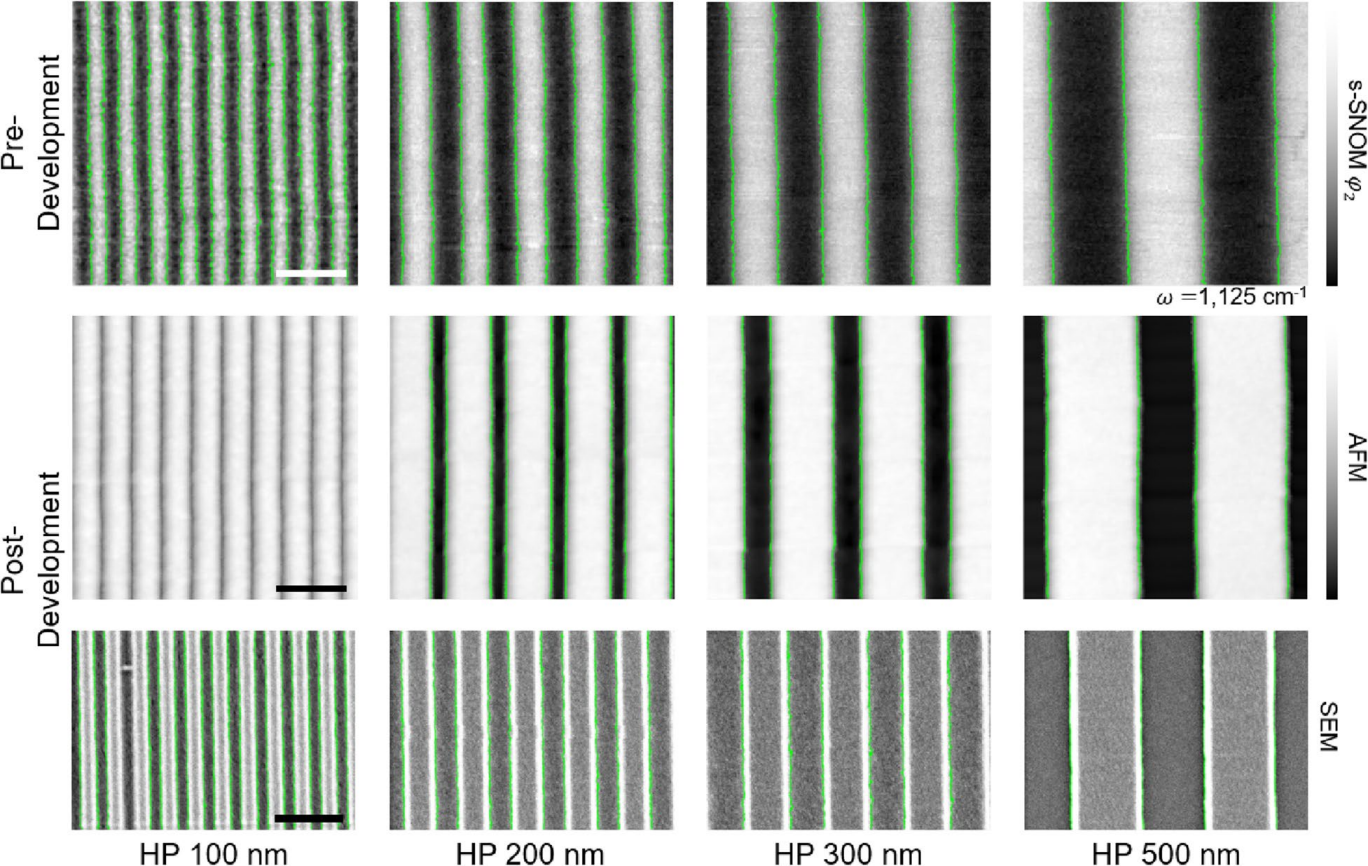
LER analysis images of HP 100, 200, 300, and 500 nm. (Top) s-SNOM results of latent images that were taken with a 1125 cm^−1^ infrared laser. (Middle) AFM topographic and (bottom) scanning electron microscopy (SEM) results of the developed pattern. Due to the 3-dimensional (3D) shape of the probe tip, the AFM could not visualize the HP 100 nm pattern. The LER values of the HP 200 and 300 nm patterns for SEM are overestimated due to the weak image contrast. The scale bar is 500 nm for all cases

To quantitatively analyze the line roughness, the s-SNOM and AFM images were converted into gray tones. For more precise analysis, detailed scans representing 2 × 2 μm^2^ areas were taken in both methods. SEM images of the developed samples were also taken, as shown in Fig. 5. The boundaries of the lines and spaces were successfully recognized in all gray-scale images; thus, the linewidth and LER information [56] could be successfully obtained. The LER is calculated as 3 $\sigma$, where $\sigma$ is the standard deviation [54]. Defined as the $\sigma$ of deviations from the ideal straight line, LER is given by

$$LER=3\sigma =3\sqrt{\frac{1}{N}\sum_{i=1}^{N}{\left({x}_{i}-\overline{x }\right)}^{2}},$$

where ${x}_{i}$ is the local position of the edge position $i$ on the line, $\overline{x }$ is the average of the points ($\overline{x }=1/N({\sum }_{i=1}^{N}{x}_{i}$), and *N* is the total number of points [57]. Note that we minimized the number of post-processes involving the s-SNOM and AFM data to prevent any unintended effects on LER. For the s-SNOM data, leveling correction and noise reduction correction were performed, while the AFM data only went through leveling correction. SEM data was not treated with any corrections.

Table 1 presents the measured linewidth and LER values. The roughness is affected by various factors, including development time, temperature, and developer concentration [58–61]. Typically, the LER value of HSQ is known to be 3–10 nm, which is similar to our AFM values of HP 200 and 300 nm [62–66]. The HP 500 nm pattern showed smooth lines like the HP 200 and 300 nm patterns, but it also had a high LER value, which was attributed to the distortion. As shown in Table 1, the LER values of overall s-SNOM were about twice as high as the AFM-measured values, except for HP 500 nm. This discrepancy may be attributable to differences in the way the signal was acquired. The optical signal of the s-SNOM is expected to have a lower signal-to-noise ratio (SNR) than the topographic signal of the AFM. However, although the two values were not the same, the fact that they differed at a similar rate implies that the pre-development inspection was valid for pattern evaluation. Therefore, it is expected that the s-SNOM can contribute to the pattern study of the latent photoresist images.

**Table 1:** Linewidth and LER values from s-SNOM, AFM, and SEM. HP 100 nm image from AFM was excluded from the evaluation because it was not fully scanned

Unit: mm	Pattern	Linewidth	LER
s-SNOM	HP-100	105.3	19.87
	HP-200	208.1	18.92
	HP-300	309.9	18.23
	HP-500	519.0	17.18
AFM	HP 100	–	–
	HP 200	315.3	8.770
	HP 300	437.0	10.82
	HP 500	639.6	15.14
SEM	HP 100	123.5	9.050
	HP 200	243.6	12.31
	HP 300	352.1	16.00
	HP 500	529.8	8.960

In addition, the s-SNOM and SEM images clearly show 1:1 lines and spaces ratios, but AFM showed thicker lines than spaces. For all patterns, the linewidths of AFM were ~ 140 nm wider than the intended design. This discrepancy may have been caused by the 3-dimensional (3D) shape of the AFM tip and the thickness of the photoresist. For this reason, in the case of HP 100 nm, lines and spaces were not fully revealed due to the interference between the pyramid-shaped AFM tip and the plateau-shaped photoresist. Therefore, even though the AFM provides clear images, it does not accurately measure the linewidths. Meanwhile, SEM often offers weak contrast images for thin films, so it is difficult to precisely measure roughness using SEM [29–33]. For example, in our results, the LER values calculated from the two clear contrast images (HP 100 and 500 nm) are almost the same as the AFM values. On the other hand, due to the unclear pattern boundaries, the LER values of blurred images (HP 200 and 300 nm) appeared to be larger than the AFM values.

By contrast, since s-SNOM measures fully flat films before the development process, it is free from issues caused by the thin film thickness and the 3D structure, so it was able to successfully take the linewidths and LER values for all cases. Further, because s-SNOM employs infrared, which has low energy and is sensitive to molecular vibrations, it provides high contrast chemical images without changing or destroying the chemical structure of the photoresists. These images and data show the potential of s-SNOM as a new inspection technique that provides pattern information of latent images that cannot be obtained using existing metrology and inspection methods such as SEM and AFM. A supplementary information page contains the experimental design and further result from the near-field infrared nanoscopic study of EUV and e-beam exposed hydrogen silsesquioxane photoresist (Additional file 1: Figs. S1–S5).

## Conclusions

In this study, to test the validity of s-SNOM in quantitative chemical analysis of photoresist materials, EUV- and e-beam-exposed HSQ films were measured by FTIR and s-SNOM. To this end, two HSQ films were patterned by focused e-beam to have HP 100, 200, 300, and 500 nm lines and spaces. One film was developed and scanned by AFM and SEM, while the other was observed by s-SNOM without any wet development processes. The patterns—including linewidth and LER—could be definitively identified in the non-developed film by s-SNOM, and this identification was validated through comparison to AFM and SEM images. While the images observed by AFM and SEM respectively had 3D structural issues and low contrast issues, the s-SNOM chemical scanning showed reliably consistent linewidths and LER values for all cases. This suggests that the s-SNOM could be a promising tool for examining the patterns formed in the photoresist films before a wet development step, which is not possible with the existing metrology and inspection tools. This spectroscopy-based imaging protocol provides chemical insight with regard to the changes occurring in the photoresist films, which is very useful information in advanced material research.

## Supplementary Information

**Additional file 1. Fig. S1.** (a) EUV dose design of HSQ film. (b) Optical microscope image of as-exposed HSQ film. (c) Film thickness. **Fig. S2.** (a) AFM topography of EUV-exposed positions and (b) their line profiles. **Fig. S3.** (a) E-beam patterning design of HSQ film. (b) Detailed design of HP pattern and SEM image. (c) s-SNOM chemical images of two patterns. **Fig. S4.** Full set of s-SNOM images for HP 300 nm pattern. **Fig. S5.** Post-processing procedure of AFM and s-SNOM images.

## Data Availability

The datasets used and/or analyzed during the current study are available from the corresponding author on reasonable request.
