# Supplementary material for: Near-field infrared nanoscopic study of EUV- and e-beam-exposed hydrogen silsesquioxane photoresist
Source: Nano Converg. 2022 Dec 2;9:53. doi: 10.1186/s40580-022-00345-3 (PMC9718909; doi:10.1186/s40580-022-00345-3)
Supplement: Supplementary file 1 — Additional file 1. Fig. S1. (a) EUV dose design of HSQ film. (b) Optical microscope image of as-exposed HSQ film. (c) Film thickness. Fig. S2. (a) AFM topography of EUV-exposed positions and (b) their line profiles. Fig. S3. (a) E-beam patterning design of HSQ film. (b) Detailed design of HP pattern and SEM image. (c) s-SNOM chemical images of two patterns. Fig. S4. Full set of s-SNOM images for HP 300 nm pattern. Fig. S5. Post-processing procedure of AFM and s-SNOM images. [file 40580_2022_345_MOESM1_ESM.docx]

*Supplementary Information*

Near-field infrared nanoscopic study of EUV and e-beam exposed hydrogen silsesquioxane photoresist

Jiho Kim^1^, Jin-Kyun Lee^2^, Boknam Chae^1^, Jinho Ahn^3*^ and Sangsul Lee^1*^

^1^Pohang Accelerator Laboratory, POSTECH, Pohang 37673, Republic of Korea

^2^Department of Polymer Science & Engineering, Inha University, Incheon 22212, Republic of Korea

^3^Division of Materials Science and Engineering, Hanyang University, Seoul 04763, Republic of Korea

^*^E-mail: jhahn@hanyang.ac.kr, sangsul@postech.ac.kr


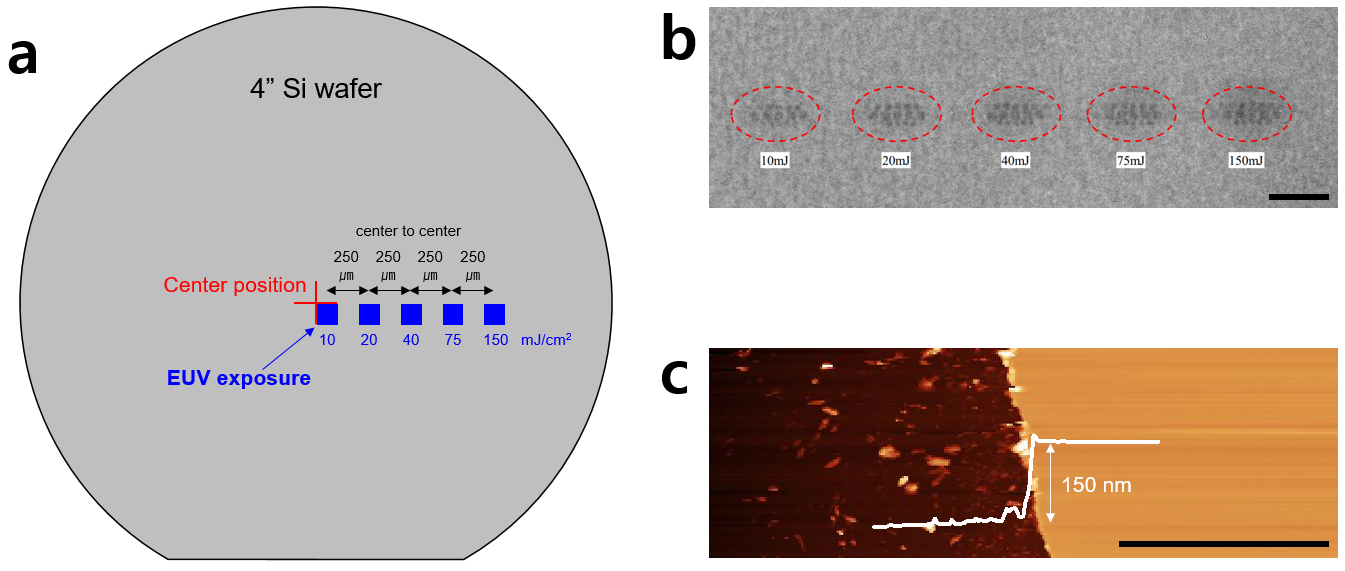


**Fig. S1.** (a) EUV dose design of HSQ film. EUV doses with energy density 10, 20, 40, 75, and 150 mJ/cm^2^ were irradiated on 150 nm thick HSQ, with 250 μm lateral spacing. (b) Optical microscope image of as-exposed HSQ film. (c) The film thickness of HSQ was measured by AFM. All scale bars are 10 μm.


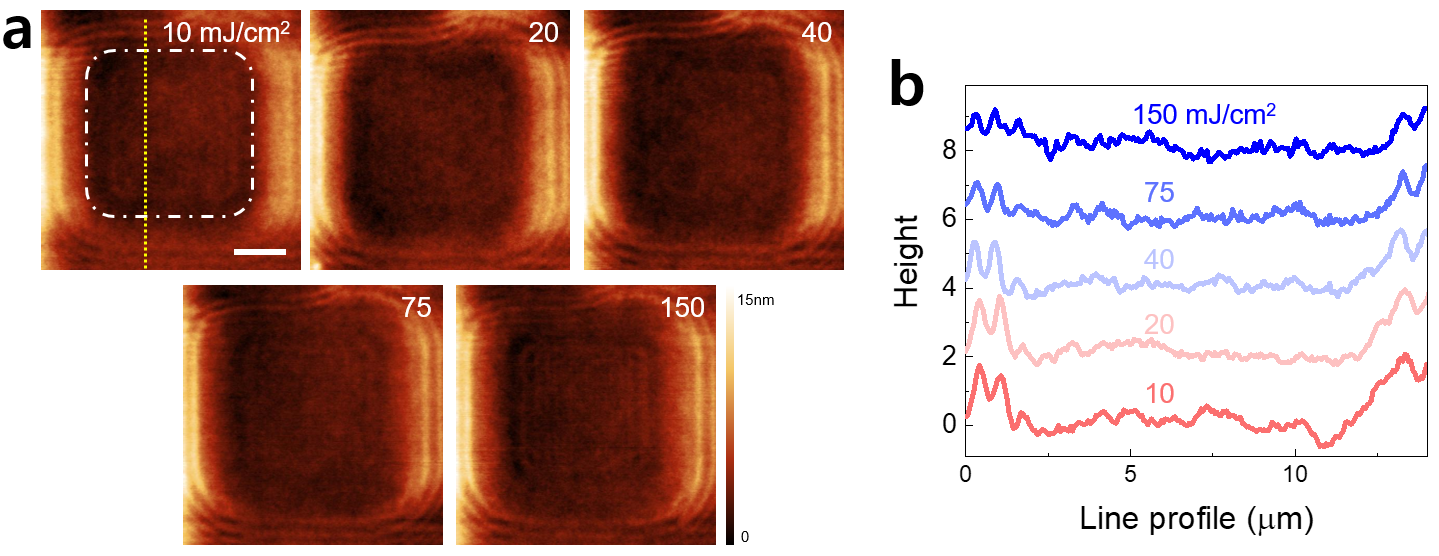


**Fig. S2.** (a) AFM topographic images of EUV exposed positions for various EUV energy. The white dash-dot line indicates the EUV exposed area. These AFM data are obtained simultaneously with s-SNOM data, which are shown in Figure 2 (a). The scale bar is 3 μm. (b) The line profile is taken from (a) as depicted by the yellow dotted line. For all data, the local thickness differences between exposed and non-exposed areas are less than 2 nm. Thus, we assumed that the thickness of all exposed positions is the same and plotted the FTIR spectra without thickness normalization in Fig. 1 (b) and Fig. 2 (c).


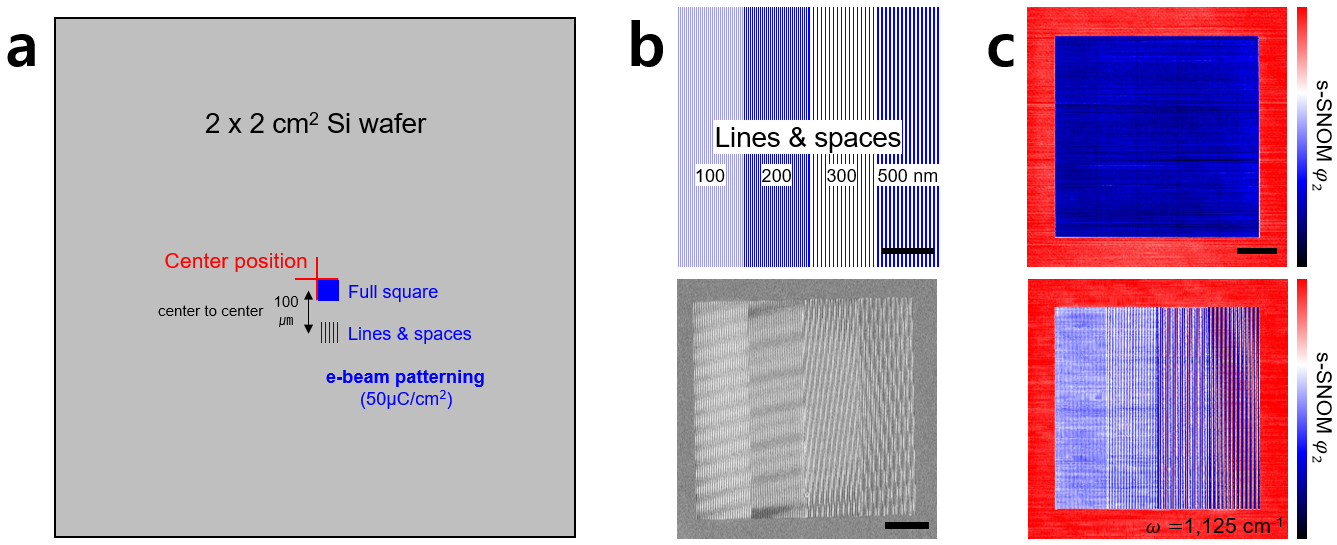


**Fig. S3.** (a) The e-beam patterning design. (b) Detailed design of (top) lines & spaces pattern and (bottom) its SEM image, respectively. Line and space patterns are not suitable for FTIR measurement because it provides the blended signal of exposed and non-exposed areas. Therefore, FTIR microscope measurements were performed at the full square pattern. (c) s-SNOM 2^nd^ order phase ($\varphi_{2}$) images of (top) full square and (bottom) lines & spaces patterns. The s-SNOM images were taken at 1,125 cm^-1^. All scale bars are 10 μm.


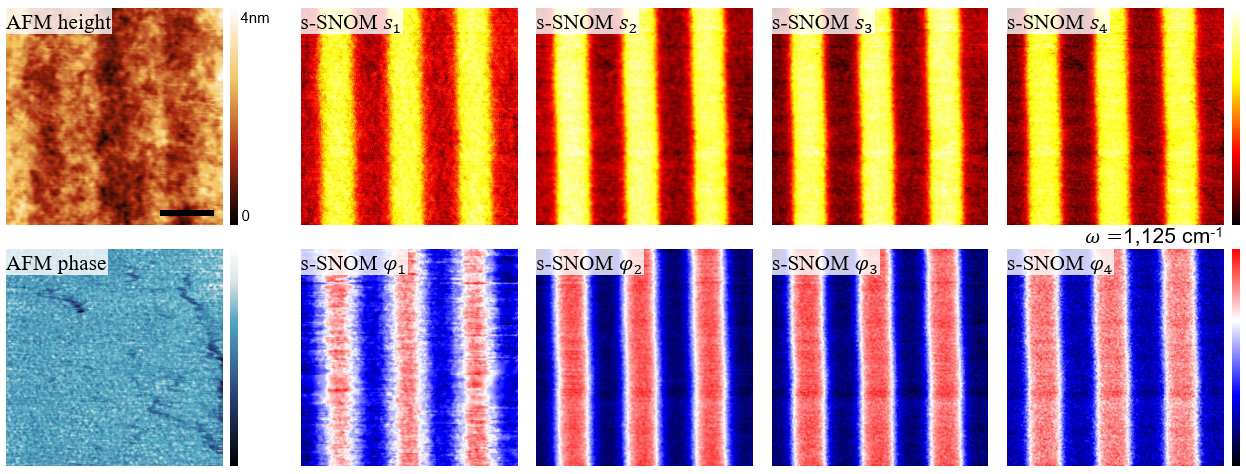


**Fig. S4.** A full set of s-SNOM images for HP 300 nm pattern. All images were recorded simultaneously. High order s-SNOM images clearly show 1:1 lines and spaces pattern design in both amplitude images ($s_{2}$, $s_{3}$, and $s_{4}$) and phase images ($\varphi_{2}$, $\varphi_{3}$, and $\varphi_{4}$), while AFM height and phase images did not. The s-SNOM images were taken at 1,125 cm^-1^. The scale bar is 500 nm.


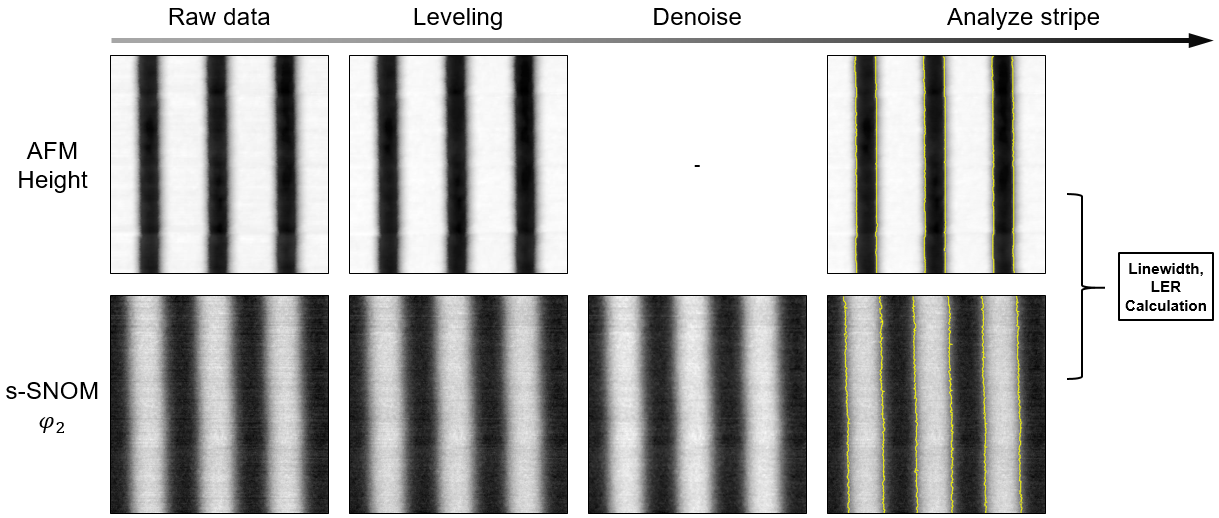


**Fig. S5.** Post-processing procedure of AFM and s-SNOM images. This example is HP 300 nm pattern. The s-SNOM $\varphi_{2}$ image was taken at 1,125 cm^-1^. To prevent artificial effects on LER calculation, we minimized the processes. In this paper, all SEM images were not treated any post-processes.
